# Exploring the challenges faced by Dutch truck drivers in the era of technological advancement

**DOI:** 10.3389/fpubh.2024.1352979

**Published:** 2024-04-24

**Authors:** Joost de Winter, Tom Driessen, Dimitra Dodou, Aschwin Cannoo

**Affiliations:** ^1^Faculty of Mechanical Engineering, Delft University of Technology, Delft, Netherlands; ^2^Transporteffect BV & Chauffeursnieuws, Nijmegen, Netherlands

**Keywords:** professional drivers, work pressure perception, safety, truck drivers, vehicle technologies

## Abstract

**Introduction:**

Despite their important role in the economy, truck drivers face several challenges, including adapting to advancing technology. The current study investigated the occupational experiences of Dutch truck drivers to detect common patterns.

**Methods:**

A questionnaire was distributed to professional drivers in order to collect data on public image, traffic safety, work pressure, transport crime, driver shortage, and sector improvements.

**Results:**

The findings based on 3,708 respondents revealed a general dissatisfaction with the image of the industry and reluctance to recommend the profession. A factor analysis of the questionnaire items identified two primary factors: ‘Work Pressure’, more common among national drivers, and ‘Safety & Security Concerns’, more common among international drivers. A ChatGPT-assisted analysis of textbox comments indicated that vehicle technology received mixed feedback, with praise for safety and fuel-efficiency improvements, but concerns about reliability and intrusiveness.

**Discussion:**

In conclusion, Dutch professional truck drivers indicate a need for industry improvements. While the work pressure for truck drivers in general may not be high relative to certain other occupational groups, truck drivers appear to face a deficit of support and respect.

## 1 Introduction

Given the Netherlands’ strategic position as a gateway to Europe and its port infrastructure, the truck driving profession plays a key role in the economic success of the country. As of 2021, approximately 91,000 professional truck drivers were registered in the Netherlands (1).

Truck drivers face various challenges that can affect their well-being, such as long working hours and extended periods away from home, which may adversely impact mental health and familial relationships (2–7). Additionally, the sedentary nature of the truck driving profession involves health risks such as obesity (8–10). Another challenge faced is the pressure to meet tight delivery schedules, which can result in fatigue and compromised road safety (2, 11–13). A study among truck drivers by Wijngaards et al. (14) showed that the driving itself, as well as the rest breaks and administrative tasks, are associated with greater momentary happiness compared to logistical work and the delivery/pickup of goods.

Truck drivers also grapple with adapting to the evolving technological landscape, including the adoption of advanced driver assistance systems (ADAS), such as adaptive cruise control (ACC) and lane keeping assistance (LKA) systems, as well as digital tools that aim to improve safety and efficiency (15). While new technologies offer potential benefits, they can also generate resistance (16), cause apprehension about job displacement (17), and require truck drivers to acquire new skills (18–20). Semeijn et al. (21), for example, reported that the digital tachograph is a source of stress.

Various studies have been undertaken on the topic of ADAS, typically using driving simulators and focusing on passenger vehicles (22–24). Current literature suggests a preference among truck drivers for a silent cabin environment (25, 26). Certain systems, such as autonomous emergency braking (AEB) and warning systems, are likely beneficial from a safety perspective (27, 28). However, these systems exhibit a propensity for false interventions/alarms, rendering them as annoying or intrusive (29–32). Camera systems and ADAS that reduce blind spots, on the other hand, have been met with approval by truck drivers (33). Still, which ADAS are perceived by truck drivers as useful and which as less useful has not yet been well documented in the literature.

### 1.1 Research aim

Although certain pain points in the trucking industry have been documented [e.g., tight schedules, stress, and fatigue (34–37)], there is still limited knowledge about how truck drivers experience their daily work. This is particularly relevant in recent years, as factors such as driver shortages (38) and the introduction of new technologies are playing increasingly large roles.

The aim of this study is to document the experiences of Dutch truck drivers. A large-scale questionnaire was conducted by Transporteffect BV (which is engaged in advisory services and mediation within the transportation sector) and foundation Chauffeursnieuws (a website focused on the transport industry). Although the results of the questionnaire have been published in raw form on the organization’s website (39), they have not yet been subjected to scientific evaluation. This paper analyzes the results of this questionnaire, which includes responses from over 3,700 drivers, through a multivariate statistical approach and through a ChatGPT-aided text summarization approach. This analysis allowed for making informed statements about the experiences of drivers and to determine whether there are relevant patterns in their experiences, which may potentially correlate with individual differences such as gender, age, and type of work (national vs. international). By better understanding truck driver experiences, policymakers and industry stakeholders could make more informed decisions to improve the working conditions and job satisfaction of truck drivers.

## 2 Methods

### 2.1 Questionnaire design

The questionnaire header indicated that Chauffeursnieuws & Transporteffect aimed to address the long-neglected concerns of professional drivers and promote their welfare. It stated that by providing a platform for drivers to voice their opinions, the organizations were committed to creating a positive impact on the transportation sector.

The questionnaire was administered in Dutch and consisted of 68 questions divided into 9 parts. It included 51 multiple-choice questions, 1 checkbox question, and 15 open comment boxes that provided the option to the respondent to elaborate on the preceding multiple-choice questions.

Part 1: Introduction (Q2–Q6) gathered general information about the respondents. Example questions included: “*Your gender?*” (Q2) with response options *Male* and *Female*, and “*Are you a professional driver?*” (Q3) with response options *Yes* and *No*.

Part 2: Organizations (Q7–Q12) focused on the respondents’ involvement and opinions on trade unions and other organizations. For example, “*Are you a member of a trade union?*” (Q7) with response options *Yes* and *No*, and “*CNV – What grade would you give?*” (Q9), with response options *1 (Very bad)* to *5 (Very good)*, and *No opinion*.

Part 3: Image (Q13–Q17) dealt with the public image of drivers and related topics. Example questions were: “*Do you think the image of the driver needs to be improved?*” (Q13) and “*Do you think a mobile toilet (DIXI) at companies is a good solution for drivers?*” (Q14), both with response options *No*, *Yes*, and *Neutral*.

Part 4: Traffic safety (Q18–Q30) explored the respondents’ views on various traffic safety issues. Example questions included: “*Do you think a stand-alone air conditioner contributes to road safety in Europe?*” (Q18) with response options *Yes*, *No*, and *No opinion*, and “*Do you find text signs with information adequate for international traffic?*” (Q21) with response options *No – creates dangerous situations*, *No*, *Yes*, and *No opinion*.

Part 5: Work pressure (Q31–Q39) investigated the respondents’ experiences and opinions about work pressure. Example questions were: “*Do you experience high work pressure?*” (Q31) with response options *No*, *Yes – every day*, *Yes – 1 or 2 times per week*, and *Yes – 1 time per month on average*, and “*Do you think work pressure should be addressed?*” (Q35) with response options *Yes*, *No*, and *No opinion*.

Part 6: Transport crime (Q40–Q44) focused on transport crime issues and their impact on the respondents. Example questions included: “*Have you dealt with transport crime?*” (Q40) with response options *Yes – regularly*, *Yes – sometimes*, and *No*, and “*Do you report all forms of crime to the authorities? Or via* https://meldpunt-transport.nl/’’ (Q42) with response options *No – small events not*, *No – never*, *Yes – only big events*, and *Yes – all events*.

Part 7: Driver shortage (Q45–Q56) explored the respondents’ perceptions of the driver shortage and related topics. Example questions were: “*Do you feel the demand for professional drivers has increased?*” (Q45) with response options *Yes – much more demand*, *Yes – a little more*, *No – not more than in the last 10 years*, and *No opinion*, and “*Do you find the hourly wage sufficient compared to similar jobs?*” (Q47) with response options *Yes*, *No*, and *No opinion*.

Part 8: General questions (Q57–Q66) dealt with various topics, including paid parking and the European Mobility Package [EU regulations to improve road transport conditions (40)]. Example questions included: “*Do you think paid parking for trucks is a solution?*” (Q57) with response options *Yes – better facilities*, *Yes – only if well organized*, *No – only take money from the sector*, *No – no rest possible*, and *No opinion*, and “*What do you think of the current European Mobility Package?*” (Q62) with response options *1 (Bad)* to *5 (Very good)*.

Part 9: The concluding section (Q67–Q68) provided space for respondents to share their opinions on the most important changes needed in the sector and any additional comments or suggestions. The two questions were: “*Open question: What is, in your opinion, the first thing that needs to change in the sector? (Please provide 1 answer)*” (Q67), and “*Comments and suggestions that you could not include in the questions can be written below.*” (Q68).

The open comment boxes were present in each part: Part 2 (Q12), Part 3 (Q17), Part 4 (Q25, Q28, Q30), Part 5 (Q34, Q39), Part 6 (Q44), Part 7 (Q51, Q53), Part 8 (Q58, Q60, Q63), and Part 9 (Q67, Q68). For an overview of all questions, please refer to the Data availability section.

### 2.2 Questionnaire dissemination

The questionnaire was administered in September and October 2021, with invitations disseminated through the website www.transporteffect.com and the corresponding LinkedIn and Facebook pages, platforms for sharing truck-related news articles.

### 2.3 Data pre-processing

In total, 3,845 respondents completed the questionnaire. Of these, 137 indicated that they were not professional truck drivers and were therefore excluded from the analysis, leaving 3,708 respondents. The questionnaire contained 51 multiple-choice items, which were analyzed separately from the open comment boxes. One question (Q15, about mobile toilets) was excluded because we considered it unclear.

The 50 remaining questions were divided into three categories:

- Driver-related questions (Q2: “*Your gender?*,” Q3: “*Are you a professional driver?*,” Q4: “*Where do you primarily drive?*” (*1: National*, *2: Benelux + Ruhr area*, *3: International*), Q5: “*How old are you?*”).
- General outcome questions (Q13: “*Do you think the image of the driver needs to be improved?*,” Q16: “*What is your general impression of the image of the professional driver?*,” Q46: “*Would you recommend the profession to family or acquaintances?*,” Q56: “*What grade would you generally give to the professional driver’s profession?*,” Q64: “*How do you see the future as a Dutch professional driver?*”).
- Forty-one, more specific, questions.

The driver-related questions and general outcome questions were used as criterion variables, while the remaining questions were subjected to a multivariate statistical analysis.

Response options for questions were not always on an ordinal scale and sometimes included *Not applicable*, *No opinion*, or *Do not know* choices. Therefore, the response options were sorted from low to high, response options that were equivalent on an ordinal scale (for example, *No – creates dangerous situations* and *No*) were combined, and the *Not applicable/No opinion/Do not know* options were marked as missing responses, since such responses cannot be used in standard linear statistical methods. For an overview of the response frequency distributions pertaining to each question, please refer to the Data availability section.

The number of times *No opinion*, *Not applicable*, or *Do not know* were answered was low for some questions (e.g., 0.2% for Q50, “*Do you think the driver’s profession gets the respect it deserves?*”). However, for some questions, these responses were more frequent. For example, for the question “*Do you report all forms of crime to the authorities? or via* https://meldpunt-transport.nl/’’ (Q42), 42.4% reported *Not applicable*, presumably because these drivers had not experienced any crime.

Regarding the grading of different unions and trade organizations (Q8–Q11), there was also a high prevalence of *No opinion* responses (26.0, 44.0, 32.8, and 24.9%), likely because drivers were not members or had not dealt with every organization. Since the aim of our research was to assess the general sentiment of drivers, not specific organizations, these four questions were combined into one by averaging, reducing the percentage of missing data for this question to 10.0%.

As the overall number of missing responses was low (6.9% of the 3,708 × 38 matrix of numbers), it was decided to impute these missing values, approximately preserving the means and intercorrelations between item responses. Specifically, missing data were imputed using the nearest-neighbor method, whereby the missing data in the 3,708 respondents × 38 questions matrix were imputed with the value of the nearest-neighbor row according to the Euclidean distance.

### 2.4 Statistical analysis

The mean scores on the 38 questions were interpreted to describe key patterns. Following this, the data (3,708 × 38 matrix of numbers) were subjected to exploratory maximum likelihood factor analysis. This statistical method aims to explain the correlations among variables by identifying latent factors that influence these variables; it is frequently used in the analysis of questionnaire data to reveal underlying psychological constructs (41). The number of factors to extract was based on the screen plot, a graphical representation where eigenvalues (corresponding to the percentage of variable explained) of the correlation matrix are plotted in descending order. The plot generally begins with a steep slope before leveling off, creating an elbow-like shape. The point at which the slope starts to level off is deemed the optimal number of factors to retain (42).

Subsequently, the factor loadings were orthogonally rotated using the Varimax method. Although it could be expected that underlying factors would correlate positively, an orthogonal rotation was chosen. This was done because we were interested in the discriminative power of the factors and their relationship with driver characteristics (rather than a ‘general positivity’ that may be expressed in multiple factors). Factor scores were calculated using the weighted least-squares method. The factor scores were subsequently standardized to have a mean of 0 and a standard deviation of 1.

The scores on the extracted factors were then correlated with the aforementioned criterion variables. Note that Q3 (“*Are you a professional driver?*”) was not used in this analysis because we only included respondents who answered *Yes* to this question; hence, this item exhibits no variance.

### 2.5 Text analysis: summaries of open comment boxes

The questionnaire contained a number of open comment boxes. Traditional methods such as content analysis and thematic analysis involve human raters examining the text for specific themes [e.g., (43, 44)]. However, these methods come with the disadvantage of subjectivity and limited reproducibility (45, 46).

Recently, large language models have emerged as a promising alternative. ChatGPT has been shown to perform well in reading comprehension and other linguistic tasks (47–50). In this paper, we will use it for two purposes: summarizing open-ended responses and extracting sentiment from responses.

In summarization applications, ChatGPT’s capabilities have been shown in various fields (51), including clinical texts (52) and news items (53). Regarding sentiment analysis, research has shown that ChatGPT can generate mean sentiment scores that correlate strongly with human sentiment ratings and with VADER sentiment analysis, an existing sentiment analysis model (54). ChatGPT has also been found to outperform humans in extracting the stance and topics of tweets (55, 56), and surpass state-of-the-art models in analyzing various types of texts such as customer reviews, social media posts, and news items (57).

We used a custom script to upload the responses for each open comment box to OpenAI’s API (GPT-4, model: gpt-4-0125-preview; date: March 2, 2024). The responses were accompanied by the following prompt: “*Please make a very very short summary of the respondents’ comments shown above, IN ENGLISH; do not enumerate*.” The parameter temperature, which determines the degree of randomness of the output, was set to 0 to yield a nearly deterministic output.

Although ChatGPT can properly handle potential gibberish responses or ‘empty’ responses such as a single character (54), we have nonetheless applied a filter whereby only text responses of 4 or more characters were included in the input to ChatGPT. By excluding extremely short responses, we ensured our sample size was more accurately represented by respondents who offered feedback.

### 2.6 Text analysis: vehicle aids and on-board computer

A key research question of this study focuses on drivers’ perceptions of technology. The responses to the open-ended question regarding vehicle aids (Q30; “*Comment: vehicle aids*”) featured numerous comments on specific assistance systems, predominantly concerning the following four types:

- Adaptive cruise control (ACC)
- Lane departure warnings (LDW)/Lane keeping assistance (LKA)
- Emergency braking/AEB
- Camera systems and smart mirrors

For the comments in Q30, we manually identified the system(s) being referred to in the comment (Appendix A). Then, for each of the systems, the corresponding quotes were fed to GPT-4, with the following prompt:

*What do the users think about the discussed system? Give a very short summary; do not enumerate.*

The same prompt was used for the responses to the open-ended question regarding the onboard computer (Q34; “*Comment: on-board computer*”).

Finally, numerical sentiment scores were generated for the comments for each of the four ADAS in Q30, through the following prompt:

*These text messages are obtained from a textbox in a questionnaire about technology in trucks. I need you to provide a single sentiment rating about the technology being discussed in the comments, from 1 (extremely negative) to 100 (extremely positive). Only report a single number between 1 and 100, rounded to two decimals. no text!*

A bootstrapping approach was adopted for this process (54, 58), where all comments per ADAS (Q30) were sorted in random order, and the mean score over 1,000 attempts was taken as an overall indicator of sentiment. The use of this method was deemed necessary because the way ChatGPT operates brings a certain randomness to the output. By averaging over a large number of repetitions under effectively identical conditions (only the order of the comments differs), a statistically reliable assessment is obtained of how ChatGPT judges the sentiment of the respondents’ texts.

## 3 Results

### 3.1 Driver-related questions

A total of 3,708 respondents were included in the study, with 3,541 (95%) identifying as male (Q2). The age distribution of the respondents (Q5) was as follows: 270 individuals aged 18–25, 969 aged 25–40, 884 aged 40–50, 1,175 aged 50–60, and 410 aged 60–75 years old. In terms of driving regions (Q4), 1,483 respondents reported being national drivers, 1,552 identified as international drivers, 666 specified driving in the Benelux & Ruhr area (i.e., Belgium, Netherlands, Luxembourg, and the Ruhr industrial region in Western Germany), while 7 respondents indicated that the question was not applicable to them.

### 3.2 General outcome questions

Respondents expressed some concerns about the image of their industry, hesitancy to recommend the career to others, and a neutral to slightly negative outlook on the future. Specifically:

- 88.1% (3265) of respondents believe the image of the driver needs improvement, 7.3% (269) remain neutral, and 4.7% (174) disagree (Q13).
- The general impression of the image of the professional driver leans toward negative, with a mean score on the scale of *1 (Very negative)* to *5 (Very positive)* of 2.62 (Q16).
- 68.7% (2,549 respondents) would not recommend the profession of a professional driver to family or acquaintances, while 31.3% (1,159 respondents) would recommend it (Q46).
- Responding to the question, “*What grade would you generally give to the professional driver’s profession?*,” the mean grade provided by respondents was 6.27 out of 10 (Q56). The most common grade was 7 (*n* = 1,006).
- Finally, the majority of the respondents have a neutral to slightly negative outlook on the future, with a mean of 4.48 on a scale of *1 (Very negative)* to *10 (Very positive)* (Q64). The most frequently selected grade was 5 (*n* = 770).

### 3.3 Specific questions: mean ratings

The questionnaire used different response options for the questions, including yes/no and scales of 1–3 or 1–5. This differentiation aimed to better match the nature of each question, and may increase respondent engagement while reducing yea-saying bias (59). However, it inhibits direct comparison of items based on their mean score.

Table 1 shows mean scores for the 38 items, with a ‘normalized mean’ column ranging from 0 to 1, which allows a clearer view of the drivers’ agreement with statements across items. The results are interpreted below on this 0 to 1 scale.

**Table 1:** Overview of the 38 items subjected to statistical analysis.

No	Question	Response options	Mean	*SD*	Mean (normalized)
Q47	Do you find the hourly wage sufficient compared to similar jobs?	1 = No, 2 = Yes	1.04	0.20	0.04
Q14	Do you think a mobile toilet (DIXI) at companies is a good solution for drivers?	1 = No, 3 = Yes	1.09	0.38	0.05
Q37	Do you ever experience intimidation from your employer?	1 = No, 2 = Yes	1.12	0.32	0.12
Q36	Have you ever been asked to commit tachograph fraud?	1 = No, never, 3 = Yes, regularly	1.24	0.51	0.12
Q24	Do you find overtaking bans on highways beneficial for road safety?	1 = No, 2 = Yes	1.14	0.35	0.14
Q50	Do you think the driver’s profession gets the respect it deserves?	1 = No, 3 = Yes	1.34	0.53	0.17
Q40	Have you dealt with transport crime?	1 = No, 3 = Yes, regularly	1.35	0.52	0.18
Q49	Do you think you will be able to perform the job until 70 +?	1 = No, 2 = Yes	1.19	0.39	0.19
Q61	Have you ever felt that you were dealing with labor exploitation?	1 = No, 2 = Yes	1.24	0.43	0.24
Q21	Do you find text signs with information adequate for international traffic?	1 = No, 2 = Yes	1.24	0.43	0.24
Q42	Do you report all forms of crime to the authorities? Or via https://meldpunt-transport.nl/	1 = No, never, 4 = Yes, all events	1.77	0.97	0.26
Q41	Has your tarp ever been cut?	1 = No, 3 = Yes, regularly	1.53	0.61	0.27
Q7	Are you a member of a trade union?	1 = No, 2 = Yes	1.31	0.46	0.31
Q62	What do you think of the current European Mobility Package?	1 = Bad, 5 = Very good	2.45	0.88	0.36
Q33	Do you think the on-board computer contributes to high work pressure?	1 = No, 2 = Yes, definitely	1.43	0.49	0.43
Q59	What grade would you give to existing paid parking spaces?	1 = Very bad, 5 = Very good	2.72	0.95	0.43
Q19	Do you feel work pressure that affects your driving behavior?	1 = No, 3 = Yes, regularly	1.87	0.71	0.44
Q31	Do you experience high work pressure?	1 = No, 4 = Yes, every day	2.36	1.16	0.45
Q57	Do you think paid parking for trucks is a solution?	1 = No, 2 = Yes	1.46	0.50	0.46
Q66	Can you make ends meet with one salary?	1 = No, 2 = Yes	1.49	0.50	0.49
Q20	Have you ever held the phone while driving?	1 = No, never, 3 = Yes, regularly	2.05	0.67	0.52
Q8–Q11	Organizations – What grade would you give?	1 = Very bad, 5 = Very good	3.10	0.94	0.53
Q32	Do you ever continue driving when you feel tired?	1 = No, never, 3 = Yes, regularly	2.08	0.69	0.54
Q54	Do you spend every day calculating to comply with driving and rest time regulations?	1 = No, 3 = Yes, it’s difficult	2.11	0.79	0.56
Q55	Do you experience problems finding a decent parking spot in time?	1 = No, 4 = Yes, every day	2.79	0.94	0.60
Q65	As a professional driver, do you prefer to be home every evening?	1 = No, I want to be on the move as much as possible, 4 = Yes	2.87	0.83	0.62
Q48	Do you find the profession you practice demanding?	1 = No, 3 = Yes it’s heavy	2.31	0.65	0.66
Q38	Do you ever exceed driving times out of necessity?	1 = No, 3 = Yes	2.32	0.82	0.66
Q23	Do you think increasing truck speed contributes to better traffic flow and safety?	1 = No, 80 kilometers is fine, 3 = Yes, 90 kilometers is ideal	2.43	0.74	0.71
Q29	Do you think the aids in vehicles contribute to road safety?	1 = No, not at all, 4 = Yes	3.15	0.63	0.72
Q26	How do you find the quality of roads in the Netherlands?	1 = Very bad, 5 = Very good	3.99	0.67	0.75
Q35	Do you think work pressure should be addressed?	1 = No, 2 = Yes	1.81	0.39	0.81
Q43	Do you think driver education is important for raising awareness?	1 = No, 2 = Yes	1.83	0.37	0.83
Q45	Do you feel the demand for professional drivers has increased?	1 = Not more than in the last 10 years, 3 = Yes, much more demand	2.72	0.58	0.86
Q52	Do you think organizations that are there for transport are doing too little?	1 = No, 2 = Yes	1.89	0.31	0.89
Q18	Do you think a stand-alone air conditioner contributes to road safety in Europe?	1 = No, 2 = Yes	1.89	0.31	0.89
Q6	Are you generally satisfied with your employer?	1 = Very negative, 4 = Positive	3.71	0.55	0.90
Q22	Do you feel that space on the roads has decreased?	1 = No, 2 = Yes	1.91	0.29	0.91

This table presents the mean score and standard deviation (*SD*) for 3,708 respondents, along with the normalized mean, which is the mean linearly scaled between the minimum and maximum possible score on the question.

Regarding workplace and road safety, the use of mobile toilets at companies received a low score of 0.05 (Q14). Overtaking bans on highways scored only 0.14 (Q24). Aids in vehicles were assigned a score of 0.72, indicating a general agreement about their contribution to road safety (Q29). Furthermore, respondents found that a stand-alone air conditioner contributes to road safety (Q18, score: 0.89). A score of 0.91 was reported for the feeling that space on the roads has decreased, indicating a universal observation (Q22).

Regarding work pressure, a score of 0.45 was observed for drivers experiencing high work pressure (Q31), with a score of 0.44 regarding the feeling that work pressure affects their driving behavior (Q19). A high score of 0.81 was obtained for the belief that work pressure should be addressed (Q35).

In terms of compensation and financial aspects, a low score of 0.04 was found for the sufficiency of the hourly wage compared to similar jobs (Q47), while a score of 0.49 indicated that nearly half of the drivers find it difficult to make ends meet with one salary (Q66).

As for work-related issues, while most drivers reported that they are satisfied with their employers (0.90; Q6) and have not experienced labor exploitation (Q61) or intimidation (Q37) from their employers, a portion of respondents reported such issues (0.24 and 0.12, respectively). Additionally, a score of 0.18 was observed for having dealt with transport crime (Q40).

When considering work-life balance, a score of 0.62 was obtained for drivers who prefer to be home every evening (Q65). On the other hand, a score of 0.66 was obtained for drivers who exceed driving times out of necessity (Q38). This points to the difficulty some drivers face in maintaining a balance between work and personal life.

In the context of infrastructure, the quality of roads in the Netherlands received a high score of 0.75 (Q26). However, drivers reported a score of 0.60 for experiencing problems finding a decent parking spot in time (Q55).

Finally, regarding the perception of the profession and industry-related organizations, a score of 0.17 was reported for the belief that the truck driver’s profession receives the respect it deserves (Q50). A high score of 0.83 was obtained for the importance of driver education for raising awareness (Q43), while 0.86 was reported for the increased demand for professional drivers (Q45). However, a high score of 0.89 was observed for the belief that organizations supporting transport are doing too little (Q52).

### 3.4 Specific questions: factor analysis

**Figure 1:**
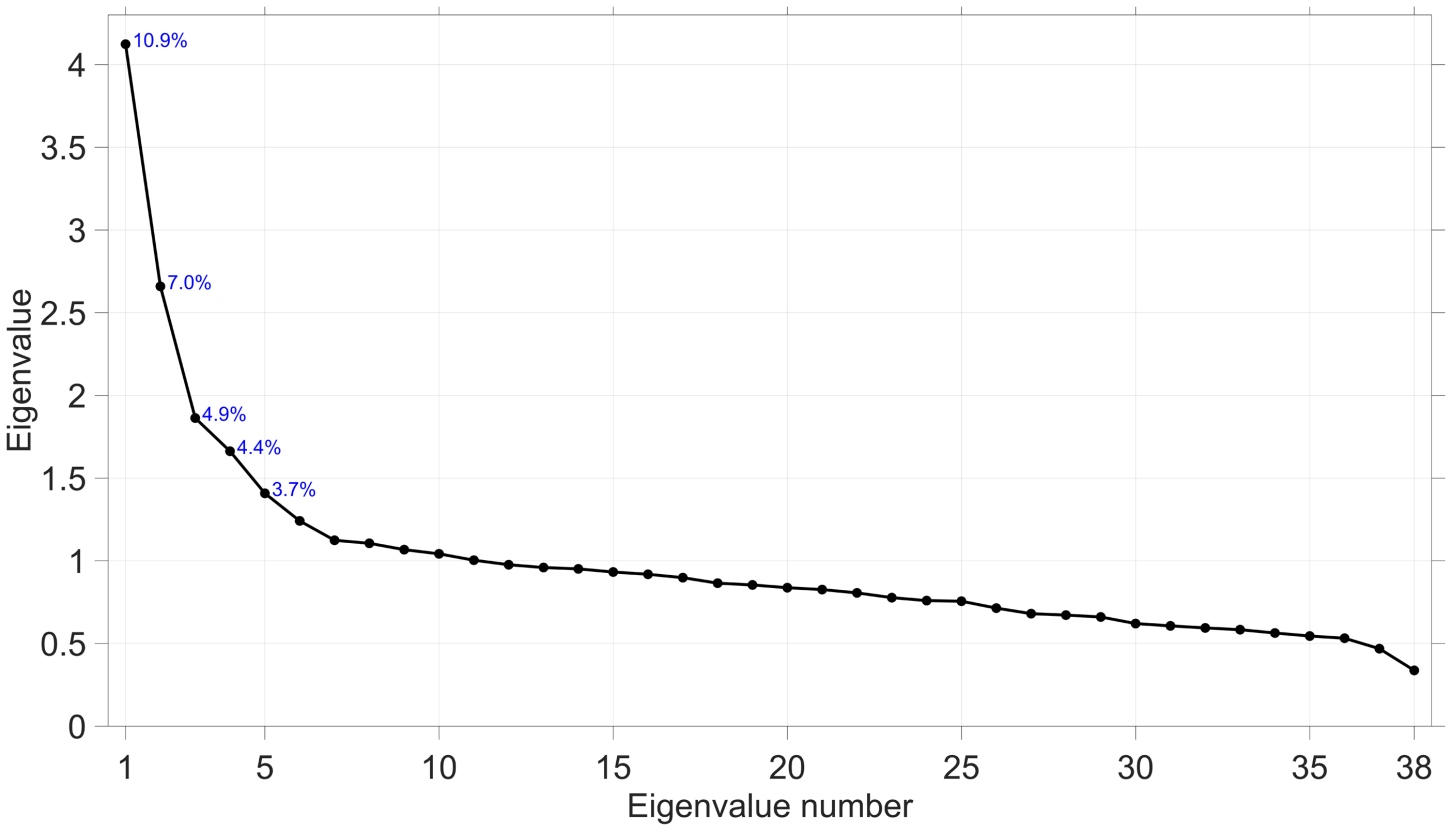
Scree plot of the 38 × 38 correlation matrix.

The results from the 38 questions were subjected to a factor analysis in order to extract underlying factors. The scree plot (Figure 1) indicated that the extraction of two factors would be appropriate, though the percentage of explained variance was not high. However, this may not impact the reliability of the constructs as long as a large number of variables correlates with the factor (60).

The Varimax-rotated loadings (please refer to the Data availability section) allowed us to interpret the two factors as follows: (1) Work Pressure and (2) Safety & Security Concerns. More specifically:

*Factor 1: Work Pressure.* Items with high loadings on this factor relate to work pressure and its impact on drivers. The strongest loadings relate to experiencing high work pressure (0.75; Q31; and 0.74; Q19). Other high loadings involve ever experiencing intimidation from one’s employer (0.45; Q37), experiencing the profession as demanding (0.45; Q48), being satisfied with one’s employer (-0.44; Q6), continuing to drive when feeling tired (0.46; Q32), the on-board computer contributing to high work pressure (0.46; Q33), and having ever felt that one was dealing with labor exploitation (0.42; Q61).

*Factor 2: Safety & Security Concerns.* Items with high loadings on this factor are related to the security and working conditions of drivers. The strongest loadings are related to dealing with transport crime (0.49; Q40), having one’s tarp cut (0.45; Q41), experiencing problems finding decent parking spots (0.51; Q55), and exceeding driving times out of necessity (0.45; Q38). Variables related to international driving showed strong loadings as well: preferring being home every evening (-0.49; Q65) and opinion about the European Mobility Package (-0.39; Q62).

The reported crimes (Q44) primarily involve diesel theft, alongside other offenses such as vehicle or container break-ins, and theft of personal belongings or cargo. Incidents of stowaways and intimidating encounters with migrants have also been noted.

Next, factor scores were calculated and correlated with the driver-related questions and the general outcome questions. The results in Table 2 show that there are small gender differences, with women being slightly more burdened by work pressure and men slightly more by crime. This latter finding can be explained by the increased likelihood of men being international drivers.

**Table 2:** Correlation coefficients between item responses and factor scores.

No	Question	Response options	Work Pressure	Safety & Security Concerns
Q2	Your gender?	1 = Male, 2 = Female	0.05	−0.09
Q4	Where do you primarily drive?	1 = National, 2 = Benelux + Ruhr area, 3 = International	-0.21	0.51
Q5	How old are you?	1 = 18–25, 5 = 60–75	-0.03	0.00
Q13	Do you think the image of the driver needs to be improved?	1 = No, 3 = Yes	0.09	0.01
Q16	What is your general impression of the image of the professional driver?	1 = Very negative, 5 = Very positive	−0.19	−0.24
Q46	Would you recommend the profession to family or acquaintances?	1 = No, 2 = Yes	-0.22	-0.17
Q56	What grade would you generally give to the professional driver’s profession?	1 = Very bad, 10 = Very good	-0.37	-0.24
Q64	How do you see the future as a Dutch professional driver?	1 = Very negative, 10 = Very positive	−0.22	-0.34

Correlation coefficients with binary variables (Q2, Q46) are also known as point-biserial correlation coefficients. Given the substantial sample size (*n* = 3,708), minor correlations statistically deviate from zero, with *p* < 0.01 when |*r*| is greater than or equal to 0.05.

The factor scores consistently correlate with the outcome measures, such as the respondents’ impression of the image of the truck driver (Q16), whether they would recommend the profession to family or acquaintances (Q46), the score they attribute to the profession as a whole (Q56), and how they view the future (Q64). Work Pressure is primarily associated with the impression of the profession now (Q56), while Safety & Security Concerns are more strongly associated with whether the future is judged optimistically (Q64).

Finally, a trend emerges wherein Work Pressure is relatively high among drivers operating nationally, while Safety & Security Concerns are relatively high among international drivers (Q4). The mean (*SD*) scores for Work Pressure are 0.22 (0.98) for national drivers, 0.05 (0.95) for drivers in the Benelux/Ruhr Area, and -0.23 (0.99) for international drivers.

**Figure 2:**
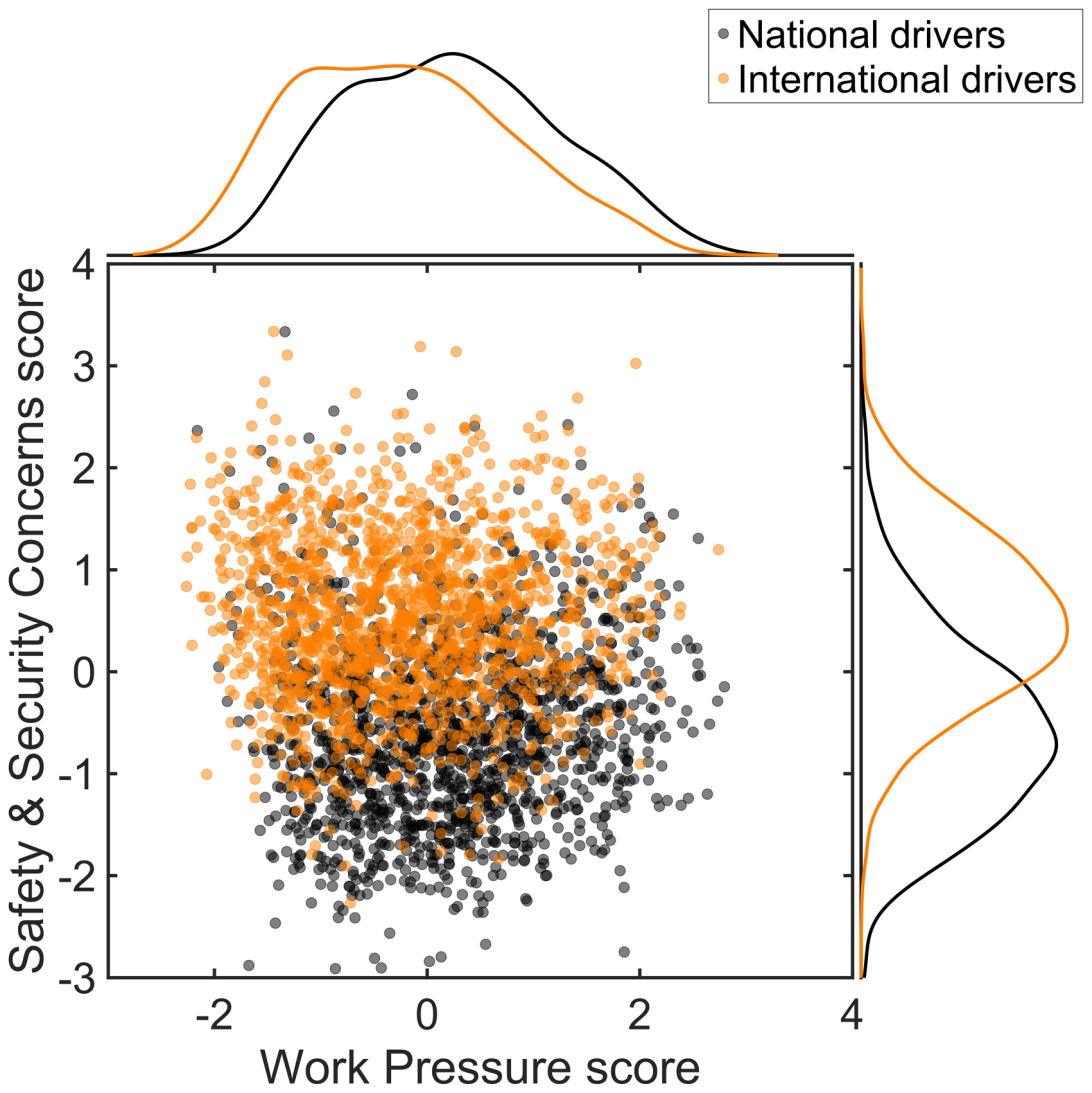
Scatter and kernel density estimate plot of factor scores, differentiating between national drivers (*n* = 1,483) and international drivers (*n* = 1,552).

On the other hand, the mean (*SD*) scores for Safety & Security Concerns are -0.57 (0.90) for national drivers, 0.00 (0.86) for Benelux/Ruhr Area drivers, and 0.55 (0.82 for international drivers. The difference in these experiences between national and international drivers is visually illustrated in Figure 2.

### 3.5 Text analysis: summaries of open comment boxes

Appendix B provides summaries of respondents’ comments regarding the 15 open-ended questions presented in the questionnaire. These summaries reveal themes that encompass a wide array of subjects, including the perceived commitment of organizations, concerns surrounding driving times, availability of parking spaces, and the contentious issue of overtaking bans for trucks. Given that the summaries of the 15 open comment boxes still contain a lot of information, and sometimes in a redundant form, we asked ChatGPT to generate a single meta-summary using the following prompt.

*Formulate a very concise summary from the following summaries without mentioning specific persons or organizations.*

The meta-summary generated by ChatGPT is as follows:

*Truck drivers express widespread dissatisfaction with their profession, citing undervaluation, mistreatment, and poor working conditions. They highlight issues such as unsanitary facilities, lack of respect from employers, clients, and the public, and the negative impact of foreign drivers. Concerns also include dangerous road conditions due to overtaking bans, inadequate education on blind spots, and mixed opinions on technological aids, which some say decrease attentiveness. Many criticize trade unions and employer organizations for not effectively representing their interests, particularly regarding wages and working conditions. The use of onboard computers is seen as increasing work pressure and invading privacy. Criminal activities like diesel theft and insufficient secure parking are major concerns, alongside the high costs and poor quality of paid parking facilities. Drivers call for improved treatment, compensation, and better enforcement of regulations to ensure fair competition and working conditions, emphasizing the need for higher wages, more flexible working hours, and better parking and rest facilities.*

### 3.6 Text analysis: vehicle aids

In addition to the general summary of the open comment boxes provided above, we conducted an analysis specifically focusing on the evaluation of technologies in the vehicle. The results of this analysis are shown in Table 3.

**Table 3:** Number of comments, ChatGPT summaries, and manually selected comments for systems mentioned in Q30 and Q34.

Category	Count	GPT-4 response and manually selected quotes
Adaptive cruise control (ACC)	74	The opinions on the Adaptive Cruise Control (ACC) system among users are mixed. Some praise it for its convenience, safety enhancements, and fuel-saving potential, appreciating its ability to maintain distance and assist in driving. Others criticize it for causing unnecessary braking, reacting to non-hazardous objects, and potentially leading to decreased driver attentiveness and reliance on technology. Concerns are also raised about its effectiveness in heavy traffic and interactions with other drivers’ unpredictable behaviors. Overall, while many see the benefits of ACC, there are significant concerns about its current implementation and impact on driving habits.
		*“Wouldn’t want to miss the ACC … anymore!”* *“Some aids also react to other situations like adaptive cruise control that also reacts to viaducts traffic signs and then it makes an emergency stop out of nowhere also extremely dangerous”* *“Certain aids are fantastic, but some also promote inattentiveness such as ACC.”*
Lane departure warnings (LDW)/Lane keeping assistance (LKA)	25	The users generally find the lane assist or line detection system to be annoying, irritating, and often unnecessary. They express frustration with false alarms and the system’s intrusiveness, with some preferring traditional driving methods without such interventions.
		*“Lane assist is mega annoying and I turn it off when I’m fit. Later in the day, it comes on.”* *“You get insanely annoyed by all those alarms. Especially from that line detection. 9 out of 10 times it goes off for no reason.”* *“Line detection … encourages you to pick up your mobile. And all that touchscreen stuff only takes the eyes off the road. Just give me press and twist buttons. The more stuff on a car the less alert people are. If something suddenly happens, people no longer know how to intervene.”*
Emergency braking/AEB	58	The users express significant concerns and dissatisfaction with the automatic emergency braking systems in vehicles, particularly trucks. They report that these systems often activate inappropriately, responding to non-hazards like traffic signs, reflections, or even shadows, leading to dangerous and unexpected braking situations. While a few see the potential safety benefits, the majority criticize the systems for creating more risks than they mitigate, especially in situations where following drivers are caught off guard by sudden stops. There’s a general sentiment that these systems need improvement to truly enhance road safety.
		*“Yes absolutely. My truck automatically brakes in an emergency situation. If it ever comes to the point where every truck has this technology (mandatory), then at least no truck will ever run into the back of a stationary traffic jam.”* *“I turn them off. Almost had an accident because the truck went full on the brakes in a slight curve at 80 km/h. The automatic braking system was triggered because my own light (headlights) reflected on a traffic sign.”* *“Some systems are downright life-threatening. For example, the emergency braking system, when you are cut off by a motorist, the system goes into action causing a great chance that your follower will shoot under your trailer.”*
Camera systems and smart mirrors	58	The users express mixed opinions about the use of camera systems in vehicles. Some appreciate the enhanced visibility and safety features cameras provide, such as reducing blind spots and aiding in maneuvers like reversing. They find cameras, including blind spot and reversing cameras, to be helpful tools that can prevent accidents. However, others raise concerns about reliability issues, such as cameras being affected by weather conditions or failing to accurately reflect depth. There’s also a sentiment that reliance on cameras can lead to decreased attention to traditional driving practices, like using mirrors and making eye contact with other drivers, potentially reducing interaction with other traffic and increasing distraction. Overall, while many see the benefits of camera systems for safety and visibility, there are significant reservations about their effectiveness and impact on driving habits.
		*“I have a camera system* etc. *for London on my car, this camera greatly reduces my blind spot and I now see much more on the highway but also on roundabouts and through cities.”* *“… Some camera systems can help. Cameras instead of mirrors, not so much, because you lose visual contact with other road users.”* *“Camera mirrors do not reflect depth and when it rains you see nothing and they break quickly.”*
On-board computer	625	The users have mixed feelings about the system, with some seeing it as a helpful tool that can make work more efficient and reduce the need for constant communication with the planning department. Others feel it increases work pressure by allowing for constant monitoring and adding more tasks, leading to a sense of being constantly watched and reducing personal freedom. Some users also mention the system can be distracting and contribute to stress, especially when it leads to additional administrative tasks or when planning uses it to push for more work to be done in less time.
		*“It depends on how the on-board computer is used. You as a driver and on the other side the planning that provides you with work. If there is good consultation with the planning, then the on-board computer is also an addition that could bring peace.”* *“You are continuously monitored, if you are ahead of schedule then extra loading addresses are added.”* *“… The on-board computer does take away the so-called “sense of freedom” although I have complete understanding for the need to account for hours.”*

ACC is valued for its safety, fuel efficiency, and calming effect on driving, though concerns about incorrect reactions and potential distractions exist. LDW and LKA systems can be irritating due to frequent alerts, leading some drivers to disable them. AEB systems can prevent accidents but raise safety concerns due to false activations, causing potential hazards for following traffic and heavy loads. Opinions on camera systems and smart mirrors are mixed; they increase visibility and reduce blind spots but can malfunction and disconnect drivers from the environment. Finally, on-board computers improve logistical efficiency and communication but may increase work pressure, create additional tasks, and infringe on driver autonomy due to real-time tracking.

**Figure 3:**
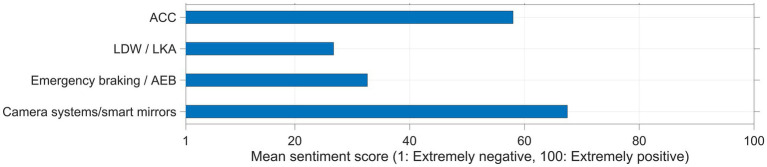
Sentiment scores for four categories of vehicle aids, as assessed by ChatGPT based on textbox comments. ACC, adaptive cruise control; LDW, lane departure warnings; LKA, lane keeping assistance; AEB, autonomous emergency braking.

The above findings are corroborated by numerical sentiment scores computed using ChatGPT. More specifically, the mean (*SD*) sentiment scores across the bootstrapped batches were 58.0 (5.07) for ACC, 26.7 (3.83) for LDW/LKA, 32.6 (4.92) for emergency braking, and 67.5 (5.14) for camera systems and smart mirrors, on a scale from 1 (*Extremely negative*) to 100 (*Extremely positive*). The reported means are shown in Figure 3.

## 4 Discussion

Truck drivers play a vital role in national distribution and international trade, yet face considerable challenges, with the rapid adoption of new technologies adding to these challenges (15, 61). However, comprehension of truck drivers’ daily experiences has been limited. The current study aimed to fill this knowledge gap through a large-scale questionnaire from 3,708 Dutch professional truck drivers. The responses to multiple-choice questions were statistically analyzed, while a large language model was used to analyze the responses to the open comment boxes.

The results revealed that Dutch professional drivers view the image of their profession as needing improvement, are hesitant to recommend it, and possess a neutral to slightly negative outlook. There was evident concern about decreased space on roads. When considering work pressure, compensation, and work-life balance, scores indicated moderate work pressure, high dissatisfaction with wages, challenges in maintaining a balance between work and personal life, and lack of support from transport organizations.

Factor analysis revealed two primary types of concerns among drivers: Work Pressure and Safety & Security Concerns. Work Pressure, characterized by high loadings on items like the impact of pressure on driving behavior and intimidation from employers, was more commonly reported by national drivers. Safety & Security Concerns, marked by high loadings on items like dealing with transport crime and finding decent parking, were more prevalent among international drivers. These results can be explained as crime primarily pertains to fuel or cargo theft when the vehicle is parked, in addition to instances of unauthorized migrants clandestinely boarding the truck (62–64). Moreover, long-distance drivers more frequently work during night hours, which may contribute to a feeling of unsafety. Work pressure was more of an issue for the national (short-distance) drivers, which may be explained by the larger number of trips they have to complete, the busier daytime traffic conditions, or the more urban traffic environments they are exposed to, in typically less comfortable vehicles (65).

In addition, our research addressed the perception of technological systems, namely ADAS and the on-board computer. ACC was appreciated for its safety features and fuel-saving properties, but concerns exist due to incorrect interventions. LKA systems were often perceived as irritating due to frequent false alerts, leading some drivers to turn them off. Some respondents saw emergency braking technology as useful in preventing accidents, but various safety concerns were raised regarding false activations [see also (29, 66)]. Camera systems and mirror technology received mixed reviews; while many respondents appreciated increased visibility and reduced blind spots [see also (33, 67)], others pointed out that the substitution of conventional mirrors with digital camesras disrupts the reciprocal visual communication between the driver and other road users, and may lead to a disconnection from the surrounding environment. Finally, on-board computers were found to improve logistical efficiency and communication but also increased perceived work pressure due to real-time tracking, potential for additional work, and a sense of surveillance. Similar concerns apply to data-driven driver coaching. Although data recorded by onboard computers has been shown to be predictive of traffic incidents [e.g., (68)], drivers may not readily accept driver monitoring systems. This reluctance could arise from drivers being unaware of the benefits or their discomfort with sharing their data with external parties (69).

These findings can be broadly interpreted in the context of automation disuse (70, 71): in general, drivers appeared to value systems that tangibly contribute to accident prevention and workload reduction, while demonstrating resistance toward less reliable systems, false alarms, and perceived intrusions of autonomy. The findings of our research can also be interpreted through the lens of Ivan Illich’s concept of ‘Tools for Conviviality’, which advocates for technology that promotes autonomy and fruitful interaction (72, 73). While features such as ACC, AEB, and camera systems can increase driver autonomy and safety when working optimally, concerns about false activations, reliability, and a sense of intrusive surveillance represent a departure from conviviality.

The sentiment ratings revealed that LDW and emergency braking yielded the lowest scores. However, these results should be interpreted with caution, as there is a possibility that drivers might have confused AEB with ACC. In recent years, ADAS have progressed substantially, typically integrating a variety of subsystems (74, 75), and their functionality may not always be clear to drivers (76, 77). Also for the authors of the current study, it was occasionally challenging to accurately classify specific comments. For example, drivers frequently referred to the term ‘distance sensor’. Technically, this is not an ADAS, but measurement equipment that is used in both ACC and AEB. This confusion may partially account for the low sentiment score for AEB, where false-positive braking interventions are typically ascribed to AEB, rather than ACC. Furthermore, for AEB, it is predominantly these false positives that drivers perceive, while the number of instances in which AEB averts accidents is logically low (78), since (near-)accidents are infrequent events. However, from a cost–benefit perspective, the AEB system might still be beneficial despite the low sentiment score, considering the substantial costs of accidents.

The acceptance of technology by drivers is essential, particularly in the context of the increasing mandating of technological systems in trucks. As of November 2015, EU regulations have made it compulsory for all new trucks to be equipped with AEB and LDW systems (79). From July 2022, new trucks are required to have additional systems, such as a blind spot information system, pedestrian/cyclist collision prevention, reversing detection, a driver availability monitoring system, and tire pressure monitoring. The mandate extends further in January 2026, when systems such as direct vision for vulnerable user protection, event data recorders, and advanced driver distraction warning systems will become obligatory (80). As more technologies become mandatory, the need for such systems to be reliable and conducive to the driver is reinforced.

Several limitations must be considered with this study. One is that the questionnaire was conducted at the end of 2021. During the COVID-19 pandemic, truck drivers dealt with less social contact as amenities closed down, while social media sentiment analysis revealed that public appreciation for their work actually grew (81).

Furthermore, it should be considered that ADAS sensors and algorithms continue to improve. While these improvements likely result in fewer false positives, there also exists the issue of human variability: false positive warnings in AEB and LDW may be inevitable considering that a threshold needs to be set for a critical time-to-collision or lateral deviation. According to the principles of signal-detection theory, this will involve a trade-off between timely warnings and false positives, as interpreted by the driver [e.g., (82, 83)]. This inescapable threshold could potentially explain why, despite many years of development, AEB and LDW systems are still perceived as irritating by drivers [e.g., (84, 85)]. Arguably, a more fundamental consideration needs to be given to the usefulness of warning systems compared to systems that automatically maintain the lane or exert torque feedback on the steering wheel (86, 87).

In this study, a large number of drivers were surveyed, which implies that the results are statistically precise. However, the results are not necessarily free of bias: it is possible that the mean values as shown in Table 1 are negatively skewed if primarily drivers who wished to complain completed the questionnaire, or if drivers exaggerated certain points in the hope that their responses would prompt a shift in national politics and business practices. In this context, it is useful to compare our results with questionnaires said to be nationally representative, specifically the National Employment Survey conducted by the Netherlands Organization for Applied Scientific Research (TNO), Statistics Netherlands, and the Ministry of Social Affairs and Employment (88). In our questionnaire, there were two questions that were highly similar to questions in this nationally representative survey. Specifically, to the question “*Do you ever experience intimidation from your employer?*” (Q37), 11.9% of our respondents answered *Yes*, compared to 10.9% in the national survey who answered *Yes* (occasionally, often, or very often) to the question “*Can you indicate to what extent you have personally experienced intimidation by superiors or colleagues in the past 12 months?*” Another comparable question was Q31: “*Do you experience high work pressure?*,” to which 19.2% of our respondents answered *Yes – every day* and 34.3% *Yes – 1 or 2 times per week* (a total of 53.5%). In the nationally representative survey, 37.1% answered *Often* or *Always* to the question “*Do you have to do a lot of work?*.” In summary, our results are in line with results from a representative sample of truck drivers in the Netherlands, suggesting no substantial bias in our questionnaire. However, it is worth noting that our open comment boxes were often left empty, with response rates ranging widely between questions (see Appendix B). It may be that drivers who wanted to suggest improvements in particular took the opportunity to fill in the open comment boxes, still introducing a form of bias.

Besides representativeness for the Dutch population, it is necessar to consider how our results relate to those of other countries. There are large national differences in road network density, road quality, accident risk, and the quality of organizations and operations. Despite this, certain factors concerning the well-being of drivers, such as stress, fatigue, and physical and mental health, recur both within Europe (13, 36, 37) and on other continents (12, 89–93).

The impression that drivers left in our questionnaire was quite negative. They appeared pessimistic about the profession as a whole and found their salary to be mediocre. At the same time, respondents were satisfied with their own employer, and the majority did not experience high work pressure, with 36.7% of respondents reporting no high work pressure and 19.2% indicating high work pressure on a daily basis. This is also evident from the aforementioned national survey, where other professional groups such as elementary school teachers, managers, cooks, lawyers, doctors, directors, social workers, and caregivers reported much higher work pressure than truck drivers (88). Possible explanations are that, even though truck drivers have many grievances about their field, ‘being on the road’ is a job that offers a certain level of satisfaction (94, 95). It is also possible that truck drivers experience pressure, but do not perceive or express it as such due to their hardship and stoicism (3). Additionally, while truck drivers may not have to work hard in physical terms, their work scheduling is highly dictated as compared to some other professions like directors, scientists, and advisors. The literature concurs that flexibility and autonomy over work hours can influence job satisfaction; a meta-analysis by Shifrin and Michel (96) highlights the positive impact of flexible work arrangements on overall job stress levels. Work-related pressures, often tied to truck driving accidents, can stem from various factors such as supervisor pressure, inadequate training, and unsupportive management (34, 36, 37). Further, loading/off-loading site culture (37, 97, 98), as well as other road users’ behavior (7, 21, 99–101), can be a source of stress.

Beyond the issue of representativeness, it is important to also monitor the quality of the responses, that is, whether the questionnaire appears to have been completed sincerely. Our impression is that the quality of the responses was high compared to other questionnaires that seem to be plagued by acquiescence bias [for discussions, see (102–104)]. An illustration of the high quality of responses is that only 3 of the 3,708 respondents (0.08%) rated the quality of roads in the Netherlands (Q26) as very bad. If there were mindless responses, the distribution of responses would be more uniform.

A noteworthy aspect of our study is that the text analysis was done automatically. Our observation is that the summaries and sentiment scores correspond to how we ourselves would summarize and rate the truck drivers’ comments. This statement is supported by a growing body of literature demonstrating that ChatGPT performs well in linguistic tasks, such as answering exam questions, labeling tweets and reviews, and analysis of sentiment (48, 50, 55, 56, 105, 106). The fact that texts were submitted to ChatGPT in Dutch rather than English is not necessarily a problem, as shown in several studies (107, 108). We agree with Mellon et al. (109) that the availability of large language models makes the use of open-ended questions in future questionnaires more attractive.

Nevertheless, there are some limitations to using ChatGPT. While ChatGPT is proficient in summarization and sentiment analysis [e.g., (47, 54, 110)], it may lack domain-specific expertise (111). Moreover, its output can be sensitive to the specific wording of the prompt (47). For these reasons, we undertook a manual classification of individual comments into the four ADAS categories (Q30). This approach ensured the sentiment scores were directly relevant to the specific ADAS under evaluation.

## 5 Conclusion

This study provided new insights into the experiences and perceptions of Dutch professional truck drivers. The findings illustrate the need for improved working conditions and support from transport organizations, as well as greater attention to safety and security concerns, especially among international drivers.

What policy recommendations arise from this research? Truck drivers often indicate that they should receive better financial compensation. However, when we consider the entirety of this work, including Appendix B, it becomes clear that the drivers are not just concerned with monetary incentives but also with recognition and respect for their profession. The current study offers various starting points that can help improve the welfare and status of drivers, including better sanitary and parking facilities. Additionally, it is recommended to act at an international level against fuel theft, break-ins, and other forms of transport crime. In the development of new technology, the minimization of perceived intrusiveness should be a key design criterion, both in a direct sense (unnecessary automated braking interventions and alarms) and in an indirect sense (perceived intrusions in work flexibility and autonomy). Although truck drivers appreciate technologies that improve safety and efficiency, the feeling of autonomy being compromised indicates a need for less meddlesome technology.

## Data availability statement

Original datasets are available in a publicly accessible repository: The original contributions presented in the study are publicly available. This data can be found here: https://doi.org/10.4121/577c120a-b5bb-4ba5-93b8-6143759d0249. Further inquiries can be directed to the corresponding author.

## Ethics statement

The study was conducted in accordance with the local legislation and institutional requirements. Written informed consent for participation was not required from the participants in accordance with the local legislation and institutional requirements. Approval for analysis of the questionnaire data was provided by the TU Delft Human Research Ethics Committee (approval number 3013).

## Author contributions

JW: Visualization, Validation, Supervision, Software, Project administration, Methodology, Investigation, Funding acquisition, Formal analysis, Data curation, Conceptualization, Writing – review & editing, Writing – original draft. TD: Writing – review & editing, Writing – original draft, Validation, Software, Methodology, Investigation, Formal analysis, Data curation, Conceptualization. DD: Writing – review & editing, Resources. AC: Writing – review & editing, Resources, Project administration, Methodology, Investigation, Data curation, Conceptualization.

## Supplementary material

Appendices A and B can be found online at: https://www.frontiersin.org/articles/10.3389/fpubh.2024.1352979/full#supplementary-material
