# Supplementary material for: Exploring the challenges faced by Dutch truck drivers in the era of technological advancement
Source: Front Public Health. 2024 Apr 24;12:1352979. doi: 10.3389/fpubh.2024.1352979 (PMC11080617; doi:10.3389/fpubh.2024.1352979)
Supplement: Supplementary file 1 [file Data_Sheet_1.docx]

**Appendix A: Vehicle Aids Comments by Category**

Table A.1 contains the responses to Q30 (vehicle aids) which were identified to contain comments about assistance systems. The responses can appear in more than one category.

Table A.1

*All responses by assigned assistance system. The responses have been translated from Dutch using GPT-4 and manually inspected for accuracy.*

| **Adaptive cruise control (ACC)** |
| --- |
| 1. "Not all tools are suitable. During cutting, short overtaking, or red LEDs (infrared) from the matrix, the ACC sometimes brakes hard with all the consequences. Here too, everyone should follow the rules with keeping distance and merging/exiting in the right way/on time (accelerate), stay behind the truck and not pass it at the last moment; signs are already at 1200/600/300 meters." 2. "I often drive on ACC, a great invention! …" 3. "ACC works reasonably, but I'm now driving a DAF that reacts to viaducts and the portals. Then it's a disastrous system." 4. "You quickly become comfortable with ACC, thinking the vehicle will handle it." 5. "It will only become cheaper when every truck is equipped with this; currently still dangerous, especially with adaptive cv …" 6. "Technology is very advanced these days.. On the truck really sanctified especially acc" 7. "ACC is not workable" 8. "ACC is dangerous" 9. "Adaptive control sometimes intervenes unnecessarily" 10. "I don't reject everything, the old-fashioned cruise control is a blessing. But nowadays you are more of a 'transport supervisor' than an independent thinking driver. No wonder you then start doing 'wrong' things, purely out of boredom. This time needs to be bridged until no drivers are needed at all. But for now, I don't find it becoming any more fun." 11. "ACC works perfectly …" 12. "Active cruise control is a good tool …" 13. "Distance holder works well, luxury cars keep creeping in between and truck brakes automatically creating a yo-yo effect" 14. "ACC is a great tool." 15. "Although I think an eye for keeping distance that doesn't work in the fog is a missed opportunity. That's exactly a moment when you need an extra tool." 16. "I am in favor of aids like ACC … but there really should not be too many of them or it will be distracting." 17. "Maybe automatic distance keeping but for everyone and blocking the phone. Really can't!!!!" 18. "Adaptive cruise control …" 19. "… ACC encourages you to pick up your mobile. And all that touchscreen stuff only takes your eyes off the road. Just give me press and twist buttons. The more stuff on a car the less alert people are. If something suddenly happens, people no longer know how to intervene." 20. "ACC mandatory …" 21. "Adaptive Cruise … ideal indeed!" 22. "… That automatic distance keeping is an improvement." 23. "Definitely adaptive cruise control" 24. "Some aids also react to other situations like adaptive cruise control that also reacts to viaducts traffic signs and then it makes an emergency stop out of nowhere also extremely dangerous" 25. "Some aids also respond to other situations such as adaptive cruise control, which also reacts to traffic signs on viaducts and then makes an emergency stop out of nowhere, also life-threatening." 26. "Wouldn't want to miss the ACC … anymore!" 27. "… adjustable speed limiter is something I use a lot," 28. "Adaptive cruise control does not belong in a truck. There are too many car drivers who cut in front of a truck at the last moment to take an exit or brake unnecessarily, causing such a system to react too abruptly. A start-stop system also does not belong in a truck." 29. "Distance keeper is good …" 30. "… enforce adaptive control at 75 meters …" 31. "active cruise control sufficient distance not a few meters" 32. "Adaptive cruise control has pros and cons. When a passer-by suddenly flashes in front of you to take the exit and your car makes an emergency stop, it's not always funny." 33. "You're either a driver or you're not. I don't like these new safety systems at all like … ACC. … I love the old-fashioned work, shifting gears myself and keeping the vehicle under control myself! And not a computer or something!" 34. "The ACC is a good example, but sometimes it reacts too well, causing the truck to brake unnecessarily from time to time. And with other vehicles cutting across at too short a distance, it's a matter of being careful." 35. "The ACC is great … " 36. "ACC is not safer than CC, ACC causes people to be less alert." 37. "Distance meter on Volvo is top." 38. "… Active cruise control is a good invention." 39. "I have the automatic distance and brake system, but it's more annoying than functional. Partly because of people who fly braking from the 3rd lane to the right to take the exit." 40. "… The Actros mp5 can largely drive itself on the highway, in a sense this certainly promotes more inattentiveness. As you gain more trust in the system with each kilometer and driving also starts to get more boring. Then people are quicker to pick up the phone." 41. "ACC works well when all trucks are equipped with it." 42. "Adaptive cruise is great …" 43. "The ACC is a very good tool for road safety and in my view also saves fuel." 44. "Ban/abolish cruise control …" 45. "ACC in combination with cruise control is a great tool." 46. "I drive with ADR and we have many aids, and especially keeping distance works well." 47. "… following systems like automatic distance keeping also make lazy. It is of course safe for rear-end collisions." 48. "ACC is handy but in rush hour you are almost standing still as traffic keeps coming in …" 49. "Is safer, especially the ACC, I think it's absolutely great." 50. "… Distance cruise control also not conducive to alertness, when you are allowed to overtake, people don't drive behind each other like elephants and you don't need it." 51. "The distance sensor … Yes, GREAT!" 52. "Had a truck with a distance keeper, etc. Sold it and bought an older one without all that stuff on it. I notice that I'm much more alert and involved in my work." 53. "Certain aids are fantastic, but some also promote inattentiveness such as ACC." 54. "Only the distance keeper of Volvo works well." 55. "Adapted cruise control, respect for each other." 56. "Distance control systems … work well, but unfortunately not every truck has it yet, so you still get them crawling in front of you and your car starts braking hard." 57. "As an aid, I actually find all options quite nice, I just find that in most cases too much is relied on, I sometimes catch myself letting it run with the flow on the ACC, which makes you a little less attentive I think, the distance is more than sufficient, but still." 58. "The ACC also reacts to the wrong things like traffic signs." 59. "Something like adaptive cruise control is a great aid." 60. "Automatic distance keeping is good." 61. "ACC; is still far from perfect, own truck performs unnecessary emergency braking at a bridge. Sensors to stay in the lane work fine." 62. "ACC for example works beautifully as long as those cars don't drive at 80 between the trucks and don't maintain a steady speed, very irritating." 63. "That adaptive cruise control is a lousy system, it brakes when it really doesn't need to. Consumes a lot of fuel and causes dangerous situations behind you because you don't notice the truck braking." 64. "Recently got … adaptive cruise control, I find it ideal, you can focus more on the actual driving itself." 65. "Certainly, that system which brakes very quickly if someone crosses in front of you or merges." 66. "I find that the distance keeper is in principle quite nice to keep distance. But I do find that we become lazy and pay less attention. On the road." 67. "ACC, my employer … has taken it off again. Not for safety but as they say for fuel consumption." 68. "I am not satisfied with … ACC, because car drivers pest test brake against trucks." 69. "You have to get used to aids like ACC. How the car then reacts when someone comes in between. There is also a difference in truck brand." 70. "Distance keeper is nothing on busy roads and in traffic jams." 71. "Have had ACC for 3 years. I find it a delight." 72. "The "assist systems" are the first thing I turn off before I drive. Very dangerous (think of the distance meter that just makes a mini emergency stop for a matrix sign or viaduct on the highway) and highly annoying and disturbing to have to listen to beeping for 10 hours." 73. "If there is little more to do, such as automatic cruise control, there is a high chance that drivers will pay much less attention to the road." 74. "Adaptive cruise control allows me to do my work much more calmly. I have never noticed that this makes me more inattentive … It is a very safe aid." |
| **Lane departure warnings (LDW) / Lane keeping assistance (LKA)** |
| 1. "Lane assist is mega annoying and I turn it off when I'm fit. Later in the day, it comes on." 2. "… lane assist always turns off." 3. "Lane detection is extremely irritating! It is disabled by many drivers! But safety for the driver is not necessary! Driver's airbag is not standard!" 4. "Lane assist is hopeless." 5. "Just look if you drive over a continuous line, it makes noise right away, you stay alert." 6. "You get insanely annoyed by all those alarms. Especially from that line detection. 9 out of 10 times it goes off for no reason." 7. "… Keep within the lines." 8. "Line detection … encourages you to pick up your mobile. And all that touchscreen stuff only takes your eyes off the road. Just give me press and twist buttons. The more stuff on a car the less alert people are. If something suddenly happens, people no longer know how to intervene." 9. "Remove lane warning …" 10. "You're either a driver or you're not. I don't like these new safety systems at all like line guard … I love the old-fashioned work, shifting gears myself and keeping the vehicle under control myself! And not a computer or something!" 11. "… Lane assist, nice, nice sound too, radio turns off from it. My driving instructor always said those white lines are as flat as possible so you can drive over them." 12. "Some roads are too narrow to keep your car strictly between the lines, the thing just keeps nagging." 13. "No opinion, bullshit lane control." 14. "Lane warning system is irritating …" 15. "… staying in your lane is useless because it often goes off due to peak hour lane driving, road works and even when the lines on the road shift. That's why most people turn off that sensor when starting. Given how narrow the peak hour lanes are, you can get a fright and hit a guardrail or the vehicle to your left. But about the phone in the car, since there was no response box. We are now obliged by the employer to call the customers half an hour before arrival. + the planning team messages us all day and personally I don't think that's okay. Driving and mobile phone don't go together." 16. "… Lane control is useful but annoying in road works due to stripes." 17. "Line control is not handy if you already drive defensively towards the right line." 18. "… Lane warning not really necessary, emergency lane lines make noise so you know you're not in the right place …" 19. "… lane detection … Yes, GREAT!" 20. "Lane … assist is also called the Facebook button. That says enough, I think." 21. "… Lane assist and other bells and whistles are distracting." 22. "Line detection hinders emergency brake system excellent but often at least 10 times a day utterly useless because it recognizes too many things as a collision distance keeper sensor works excellently well but only if you want to drive in a train all day." 23. "… Sensors to stay in the lane work fine." 24. "You get completely crazy from line protection." 25. "Line detection useless …" |
| **Emergency braking / AEB** |
| 1. "It often creates dangerous situations itself, for example when the emergency brake is activated. When nothing happens in front of you." 2. "It will only become cheaper when every truck is equipped with it, now it is still dangerous especially with … emergency stop." 3. "It also creates dangerous situations, especially the brake assist." 4. "It occasionally brakes automatically because of traffic signs above the highway, extremely dangerous if you are driving behind it and do not have those systems. Always looking out of the window works better." 5. "Those systems that brake automatically seem safe in terms of head-to-tail collisions, but my experience teaches me that these systems create very unexpected situations that an experienced driver would never create!" 6. "I don't always find the braking system safe." 7. "The emergency brake system sometimes activates for a sign, unfortunately." 8. "Brake assist often jumps in when I have the situation completely under control, while the (dumb) system has a different opinion." 9. "The automatic braking system on new trucks IS LIFE-THREATENING. It is often wrongly adjusted." 10. "I am in favor of aids like … automatic braking in danger but there really should not be too many of them or it will be distracting." 11. "If it works well yes personally I have often sat on an empty highway with my face on the windshield because the emergency braking system saw ghosts." 12. "Emergency brake that goes off or responds while there is nothing wrong." 13. "I turn them off. Almost had an accident because the truck went full on the brakes in a slight curve at 80 km/h. The automatic braking system was triggered because my own light (headlights) reflected on a traffic sign." 14. "The distance radar at DAF works fantastically but I still had an accident with it at the Breda ramp I was driving on the A27 at Breda/navel we were only driving 60, merging traffic was holding up the works and at the next ramp a massive amount of mergers but on the main road it went a bit faster again so I moved to the left to make room for the mergers suddenly a black Audi in front of me was cut off by a colleague Audi in front of him the collision radar intervenes but they keep driving I couldn't cancel the stop action by giving more gas and the combination kept braking until stopped by 3 cars behind so it doesn't work flawlessly." 15. "The emergency brake that activates as soon as you get cut off or it sees a cyclist on the bike path as an oncoming vehicle, works counterproductively at such a moment." 16. "… self-braking vehicle ideal indeed!" 17. "Distance sensor is a great thing if it works properly, but if it reacts at random to things that aren't there and then suddenly brakes the car, you are behind the wheel with a heart sinking feeling, let alone the person driving behind you. And why doesn't that thing work in fog or bad weather?" 18. "The emergency brake intervenes so often unnecessarily that it would actually be better to remove it …" 19. "Emergency brake system sometimes overly sensitive." 20. "I wouldn't want to miss the … emergency brake anymore!" 21. "I experienced it once when a motorist came to drive in front of me and hit his brake. Very briefly but long enough to ensure that I was nose against the windshield because the truck made an emergency stop on its own. Life-threatening, there might be snow on the ground. And the truck also does this sometimes when I drive on cruise control and there comes a portal or viaduct which it thinks is too low and bam full brake on the highway really dangerous." 22. "Auto brake assist on inner city roads and in the city very bad system." 23. "Direction warning systems are horrible, it sees a sign or a car that needs to take the exit and has to brake hard due to a sharp turn and the system intervenes by braking fully, another behind you never expects this and is then helpless for a collision which is then inevitable, the truck is only 5 months old." 24. "Near accident, self-braking system or whatever it's called, often brakes by itself when there's nothing wrong. If at that moment another vehicle is too close to me, it can end badly …" 25. "It's all nonsense oh the technical gadgets that brake for me." 26. "AEBS needs to be improved." 27. "… The automatic brake sometimes has problems with an airplane or something, as it sometimes reacts when it's not necessary." 28. "My emergency brake system intervenes at the strangest moments. At a tree viaduct or parked car." 29. "If it works well, not like with many cars from DAF that the emergency braking system intervenes in places where it is not needed." 30. "Car braking system does not always work as desired" 31. "My Actros mp4 has an emergency brake, fortunately, I haven't needed it yet, however, it is sometimes activated by matrix signs or bridges in rainy weather. Also at highway exits that lie in a curve, then it seems to the camera as if the braking car is still in front of you …" 32. "The braking system when someone suddenly crosses in front." 33. "When motorists cross too short in front of you, the emergency brake system reacts and the truck slows down with full force so that the driver behind (overtaking ban) almost runs into it …" 34. "Some systems are downright life-threatening. For example, the emergency braking system, when you are cut off by a motorist, the system goes into action causing a great chance that your follower will shoot under your trailer." 35. "… brake assist is also called the Facebook button. That says enough, I think." 36. "The anti-collision system is very irritating during twilight or at night with viaducts." 37. "I regularly encounter interventions by the automatic braking system due to reflections and shadows from a 2019 DAF." 38. "I often find them dangerous because they see things that aren't there and then intervene, like the distance/braking system that intervenes while there's nothing wrong." 39. "… the AEBS system work well, but unfortunately, not every truck has them yet, so you still get people cutting in front of you and your car starts to brake hard." 40. "I find the emergency braking system handy, but if the person behind you doesn't have it, they'll crash into you, so I have mixed feelings." 41. "I once turned off the assistance systems. Because my tractor slammed on the brakes in a curve. Even though I had plenty of space. If I had steel plates loaded at that moment. I'm sure they would have come out." 42. "That automatic braking system can be useful, but not when you're in a bend and it mistakes a traffic sign for a car and thinks you're going to have a collision and so suddenly goes full on the brakes!" 43. "I have so many beeps now, I turn them off nowadays. Imagine what it does to me when I get a warning at every viaduct at night that I'm driving towards a traffic jam and the truck goes into emergency braking. If that really happens, I instinctively step on the gas." 44. "That the vehicle itself intervenes when a car cuts you off is terrible." 45. "The automatic braking system can activate if a car suddenly shoots in front of you, and that can lead to dangerous situations." 46. "Emergency brake … super 👍" 47. "Emergency brake can also be extremely dangerous since the car stops almost instantly." 48. "Emergency brakes that respond to matrix signs are not really beneficial." 49. "Yes absolutely. My truck automatically brakes in an emergency situation. If it ever comes to the point where every truck has this technology (mandatory), then at least no truck will ever run into the back of a stationary traffic jam." 50. "Automatic braking not always because you are also dependent on other traffic and freight." 51. "AEBS can be dangerous if someone, while braking, wants to merge in front of you quickly." 52. "Recently got emergency brake system … I find it ideal, you can focus more on the actual driving itself." 53. "Emergency brake system sometimes sees the strangest objects as a danger." 54. "I am not satisfied with emergency brake … because car drivers pest test brake against trucks." 55. "Emergency brake system is abused by merging traffic, this is because people suddenly forget that as merging traffic they need to adjust their speed to the traffic on the main lane, and not serve as an obstacle causing the emergency brake system to regularly intervene resulting in rear-end collisions on the main lane and the culprit can suddenly speed off." 56. "… AEBS can be dangerous when it brakes for a turning car or matrix signs, which happens quite regularly with my DAF. In that respect, paying attention is much more accurate …" 57. "Some of these aids assume an ideal situation where all road users behave impeccably. But if, for example, a motorist wants to take the exit at the last minute and shoots across your grill, you have to be lucky that the emergency stop doesn't kick in and you're hanging with your seat belt." 58. "The 'assistance systems' are the first thing I turn off before I drive. Very dangerous (think of the distance meter that just makes a mini emergency stop for a matrix sign or viaduct on the highway) and highly irritating and disturbing to have to listen to beeping for 10 hours." |
| **Cameras and smart mirrors** |
| 1. "Those crazy cameras are also sensitive to interference and when it's dark they're an annoying light source, which just creates another blind spot." 2. "Very much so, a backup camera and a front camera." 3. "I have worked with a backup camera and side camera that automatically turn on, or can be turned on when you choose to do so. Works great!" 4. "Camera system and signals for when someone is on your left or right …" 5. "Standard equipment for all brands, cameras included, nothing more expensive." 6. "As soon as possible, cameras with sound signals, and when you look in the mirrors, there should be a warning light if there's something next to you." 7. "only cameras and no mirrors is not good, the outside world then has no idea whether the driver can see you or not, the camera must serve as an expansion of the field of view" 8. "… Some camera systems can help. Cameras instead of mirrors, not so much, because you lose visual contact with other road users." 9. "You start to rely on it, you think there's a buzzer when I cross the line. Almost don't look around anymore." 10. "In the past, there have been various cameras, blind spot mirrors, alarms for swinging out on the cars, and it doesn't work ideally with swinging out, an alarm goes off and when you look there are bushes, after a number of times this causes irritation, you get distracted by it and therefore you don't pay attention to the important things." 11. "Cameras help." 12. "Because of my cameras, I look less in my mirrors." 13. "Camera mirrors do not reflect depth and when it rains you see nothing and they break quickly." 14. "I have driven for a while with a front and rearview camera … when reversing I didn't have a front mirror or I had to keep pressing buttons during manoeuvring in a narrow busy street to switch between front and back." 15. "Camera or sensors work well, in city distribution a window in the door." 16. "I have a camera system etc. for London on my car, this camera greatly reduces my blind spot and I now see much more on the highway but also on roundabouts and through cities." 17. "Cameras all around really help." 18. "Camera works quite well but it's still the driver who drives." 19. "… This is also distracting: … 360-degree camera, reversing camera, blind spot camera." 20. "Cameras." 21. "For example, a camera behind the car is top," 22. "Camera makes a difference." 23. "Mandate cameras. Both downwards and backwards." 24. "Cameras can certainly help." 25. "Camera." 26. "I once had a car in my blind spot that I didn't see, but the system in the Volvo started beeping when I turned on the right turn signal, that prevented an accident. What I see in passenger cars can also be in trucks, that there's a light in the mirror if something is driving on your right or left …" 27. "More cameras all around." 28. "You're either a driver or you're not … Camera instead of mirrors also not safe! See me, see you is then not applicable. I love the old-fashioned work, shifting gears myself and keeping the vehicle under control myself! And not a computer or something!" 29. "Nowadays everything has to be done with a camera, I find a blind spot mirror for the front and the passenger side more than sufficient." 30. "Light in the mirror when someone is driving on the right is an important thing." 31. "More cameras for all-round visibility." 32. "Blind spot camera standard!" 33. "I have a camera for the blind spot and reverse, ideal." 34. "Camera is a good tool." 35. "Camera system instead of mirror has increased the blind spot along the cab, still need a solution for this, otherwise satisfied with the camera system instead of mirrors due to a wider field of view." 36. "Cameras for blind spot are perfect." 37. "Camera is good …" 38. "I am strongly against camera instead of mirrors. I teach my children "if you see the driver, the driver sees you" With the cameras on for example the Mercedes it's not possible to make eye contact via mirror or camera." 39. "… Yes, GREAT! Blind spot sensors and cameras too!" 40. "A camera would be handy, but I don't have it myself." 41. "Reversing camera." 42. "Car kit or camera for blind spot." 43. "As mentioned above the camera on the back for reversing." 44. "5 mirrors + camera should be sufficient, right?" 45. "Only camera for blind spot …" 46. "You now get cameras outside with screen inside so you don't have to look outside which neglects interaction with other traffic." 47. "By aids, I mean backup cameras, and for example, for the blind spot …" 48. "Reversing camera. Dashcam because of annoying car drivers." 49. "Cameras." 50. "Also causes more distraction constantly looking in cameras." 51. "Camera systems, reversing sounds, certainly help." 52. "Cameras 360 and back separately." 53. "Camera for blind spot works." 54. "When it's busy on the road, in the built-up area, a camera is really a must." 55. "Reversing camera mandatory on every vehicle and trailer." 56. "No cameras, however easy; because it happens outside.." 57. "… Cameras are handy in large vehicles because you have more visibility and can act on that yourself instead of a sensor that detects something and then just starts braking." 58. "I have already driven in a truck with monitor mirrors in the cabin, these distort and when it rains the camera outside closes and you see very little. (dangerous for safety, abolish immediately)" |
|  |

**Appendix B: Summaries of the 15 Open Comment Boxes**

The questionnaire contained 15 open comment boxes that provided the opportunity to give comments. The comments were automatically summarized with ChatGPT's API:

***Q12. Organizations (n = 506, 13.6%):*** The respondents express a range of negative sentiments towards trade unions and the TLN (Transport and Logistics Netherlands), criticizing them for not effectively representing or supporting truck drivers. Many feel that these organizations prioritize their own interests or those of employers over the needs of the workers. There's a sense of dissatisfaction with the lack of action or improvement in working conditions, wages, and respect for drivers. Some mention specific grievances such as inadequate representation during negotiations, failure to address the influx of cheaper foreign labor, and a general disconnect from the realities of the trucking profession. A few respondents also mention personal experiences of receiving little to no assistance when facing workplace issues. Overall, there's a strong sentiment that these organizations have lost touch with the drivers they are supposed to represent and have not done enough to advocate for better conditions in the transport sector.

***Q17. Image (n = 490, 13.2%):*** The respondents express a strong sentiment of being undervalued and mistreated in their profession as truck drivers. They highlight issues such as poor sanitation facilities, particularly the use of mobile toilets (Dixis), and a lack of respect from employers, clients, and the general public. Many drivers feel they are treated as inferior, facing harsh working conditions and inadequate appreciation for their role in the supply chain. The influx of foreign drivers, particularly from Eastern Europe, is frequently mentioned as contributing to a negative image and working conditions for drivers. There is a call for better treatment, respect, and working conditions for truck drivers.

***Q25. Overtaking bans for trucks (n = 1334, 36.0%):*** The respondents express strong opinions against the current overtaking bans for trucks on highways, arguing that such restrictions lead to dangerous situations and increased traffic congestion. Many feel that the bans create long lines of trucks ("treintjes") that make merging and exiting more difficult and dangerous for all road users. Some suggest that overtaking should be allowed, especially outside of peak hours or on roads with three or more lanes, to improve traffic flow and safety. There's also frustration about the lack of enforcement on those who ignore the bans, particularly foreign truck drivers. Additionally, some respondents believe that the speed difference between trucks and passenger cars is now minimal due to speed limit changes, making the bans unnecessary. Overall, there's a call for reevaluation of overtaking bans to enhance road safety and efficiency.

***Q28. Blind spot (n = 761, 20.5%):*** The respondents emphasize the importance of increased awareness and education regarding the blind spot issue around large vehicles, particularly for schools and during driving lessons for all types of licenses. They suggest that more attention should be given to teaching both children and adults about the dangers of blind spots. Many advocate for the use of technology such as cameras and warning signals to help mitigate blind spot accidents, while others believe that better mirror adjustment and driver vigilance are key. There's a consensus that stickers, like those mandated in France, are ineffective and that a combination of education, technology, and possibly regulatory changes (such as making certain driving behaviors around trucks illegal) could help reduce blind spot-related accidents.

***Q30. Vehicle aids (n = 441, 11.9%):*** The respondents' comments reflect a mix of opinions on the use of technological aids and safety systems in trucks. While some find features like adaptive cruise control and rear-view cameras beneficial for safety and convenience, others express concerns that an over-reliance on these systems can lead to decreased attentiveness and potential danger, especially when systems malfunction or react unexpectedly. There's a sentiment that too many aids can make drivers complacent, relying too much on technology rather than their own skills and awareness. Additionally, some suggest that more education for all road users, not just truck drivers, would improve safety. Overall, there's a call for a balanced approach that leverages technology without undermining the role of the driver's judgment and attentiveness.

***Q34. On-board computer (n = 625, 16.9%):*** The respondents' comments reflect a range of opinions on the use of onboard computers (BC) in the transportation industry. Many express concerns about increased work pressure and surveillance, feeling constantly monitored by their employers. Some mention the BC as a mere tool for hour registration, lacking added value, while others see it as a source of stress, especially when it leads to additional tasks or constant communication from planners. A few respondents appreciate the BC for its efficiency in handling administrative tasks and providing clear job instructions. However, the overarching sentiment is that BCs contribute to an impersonal work environment and can exacerbate work pressure, with the quality of the planning and company culture playing significant roles in how the technology impacts the driver's experience.

***Q39. Driving times (n = 722, 19.5%):*** The respondents express significant concerns about the difficulty in finding parking spaces, with many mentioning that parking areas are often full early, leading to stress and sometimes forcing them to park in unauthorized areas. There's a consensus that there are too few parking spaces available, especially after 17:00, and that this issue is exacerbated by the presence of trucks from Eastern Europe occupying these spaces. Some respondents suggest that minor infractions of the driving hours regulations should not result in high fines, especially when the alternative could pose a greater danger. The complexity and strictness of the European driving hours regulations are also highlighted, with many calling for more flexibility to accommodate the realities of traffic and delays. Stress from trying to adhere to these regulations, along with the digital tachograph, is a common theme. There's a feeling that the current system increases pressure on drivers, with some admitting to taking more risks when they are close to exceeding their driving hours. The lack of parking spaces is seen as a significant contributor to this stress, with many advocating for an increase in safe and accessible parking areas for truck drivers.

***Q44. Transport crime (n = 316, 8.5%):*** The respondents express frustration and concern over transport-related criminal activities, such as diesel theft and vehicle break-ins, highlighting a perceived lack of police presence and response, especially at night and on parking lots. They mention the scarcity of secure parking spaces and the need for better surveillance and lighting. Some suggest that reporting incidents feels futile as there's a belief that authorities do not take adequate action. There's also a call for more awareness and preventive measures, including secure parking and better education on avoiding theft. A few respondents note their specific strategies for minimizing risk, like choosing safer parking locations or using physical deterrents. Overall, there's a sentiment of dissatisfaction with the current state of security and support for transport workers.

***Q51. Respect (n = 623, 16.8%):*** The respondents express a strong sentiment of feeling undervalued and disrespected in their profession as truck drivers. They highlight issues such as being treated poorly by companies and the public, facing long wait times without explanation, and being compared unfavorably to drivers from Eastern Europe. Many feel that their work is not appreciated despite the essential role they play in the economy, noting that without their services, many goods would not reach their destinations. There is a call for better treatment, higher wages, and more respect for the profession, emphasizing that the current conditions are not sustainable or fair.

***Q53. Organizations' commitment (n = 497, 13.4%):*** The respondents express dissatisfaction with organizations representing truck drivers, feeling that their needs and concerns are not adequately addressed. They mention issues such as inadequate support during times of personal crisis, insufficient action to make the profession more attractive to younger generations, and a perceived bias towards employers over employees. There's a call for more human treatment, better representation during negotiations, and more visible actions like strikes to demand better conditions. Some feel that organizations are more focused on their own interests rather than genuinely supporting drivers, with specific frustrations around low wages, lack of parking and sanitary facilities, and the need for better enforcement of existing regulations. There's a sense of abandonment and a desire for more effective advocacy and tangible improvements in their working conditions.

***Q58. Paid parking (n = 735, 19.8%):*** The respondents express strong dissatisfaction with the current state of paid parking for trucks, highlighting issues such as high costs, lack of services, poor sanitation, and inadequate security. Many emphasize the need for better-regulated parking areas with essential facilities like restaurants and clean restrooms. There's a common sentiment against the financial burden falling on drivers, suggesting that employers or clients should cover parking costs. The lack of sufficient parking spaces, especially in the Netherlands, is also a concern. Some suggest that parking fees should be refundable through meal purchases at associated restaurants. Overall, there's a call for improved parking infrastructure and services, with costs not being passed onto drivers.

**Q60. *Would you like to see changes in existing paid parking spaces? (n = 3080, 83.1%*):*** The respondents' comments focus on the need for improvements in parking facilities for truck drivers in the Netherlands. Key points include the desire for lower or no parking fees, better and cleaner sanitary facilities, the availability of decent eating options, and the provision of more parking spaces, including specific areas for ADR (hazardous materials) parking. There is also a call for better security measures, including surveillance and fencing, and the suggestion that parking fees should be covered by employers or compensated through meal purchases. Additionally, respondents express a need for more amenities such as WiFi, healthy food options, and leisure facilities. Overall, there is a strong demand for higher quality, more affordable, and better-maintained parking facilities that cater to the needs of truck drivers.

***Q63. Mobility Package (n = 1510, 40.7%*):*** The comments express a wide range of frustrations and concerns from drivers regarding the current state of the transportation industry in Europe. Key issues include the lack of enforcement on existing regulations, particularly regarding cabotage and the exploitation of drivers, especially those from Eastern Europe. Many respondents feel that there is an unfair competition and that the regulations coming from Brussels are either ineffective or not properly enforced. There is a call for more parking spaces with better facilities, and a significant concern about the working conditions and pay of drivers. Some comments also mention the need for more uniform rules across European countries and better control of fraud and exploitation within the industry. Overall, there is a sense of dissatisfaction with the current state of affairs, with many feeling that the needs and well-being of drivers are being overlooked.

**Q67. *What is, in your opinion, the first thing that needs to change in the sector? (Please provide 1 answer) (n = 3234, 87.2%):*** The respondents' comments primarily focus on the need for improved treatment and respect for truck drivers, higher wages, and better working conditions. They express a desire for a higher basic salary, reduced taxation on overtime, and more flexible working hours to reduce work pressure. There is a strong call for equal pay and conditions for all drivers, regardless of nationality, to address unfair competition from lower-paid drivers from Eastern Europe. Additionally, respondents highlight the need for better parking facilities and rest areas, as well as a reduction in excessive regulation and fines. Overall, there is a clear demand for greater appreciation of the truck driving profession, with financial and working condition improvements to make the sector more attractive to new entrants.

***Q68. Comments and suggestions that you couldn't include in the questions can be written below (n = 559, 15.1%):*** The respondents' comments reflect a range of concerns and suggestions from individuals likely involved in the transportation and trucking industry. Key themes include the need for better pay and working conditions, frustration with strict regulations and excessive fines, and a desire for more respect and appreciation for the profession. Many express concerns about the impact of foreign drivers on the market, suggesting that tolls or fees should be implemented for foreign trucks to level the playing field. There's also a call for better parking facilities and rest areas for drivers, as well as suggestions for improving the overall image of the profession to attract new drivers. Some respondents also mention the need for more consistent and fair enforcement of rules across Europe, and a few suggest changes to retirement age and pension arrangements to better reflect the demands of the job.

**Q60 and Q63 were inadvertently set as mandatory for either the entire survey period or a portion of it, which likely explains their relatively high response rates. Note that Q67 also has a high response rate, but this question was not mandatory. A likely explanation for this is that Q67 is a generic closing question.*
